# The humanization of care after the COVID-19 pandemic in Italian historical hospitals: Martina Giusti

**DOI:** 10.1093/eurpub/ckac131.288

**Published:** 2022-10-25

**Authors:** M Giusti, C Cosma, S Stefania, N Persiani

**Affiliations:** 1 Experimental and Clinic Medicine, Università di Firenze, Florence, Italy; 2 Postgraduate Medical School in Public Health, Università di Firenze, Florence, Italy

## Abstract

Globally, the reforms of healthcare systems aim to bring back the patient at the centre of these organisations after the issues related to the COVID-19 outbreak. The patient returns to be fully considered, as an individual whose must be protected physical and psychological health as well as social well-being. Humanization of care is returning to the foreground. For centuries, art has been used throughout Europe in the health context for its power to support patients in their disease. Today, this approach can be rediscovered in historical hospitals, where tradition, art and assistance coexist. This study aims to investigate the interest for the development of projects for the humanization of care exploiting the artistic heritage of the historical hospitals owned by Health Authorities. The cross-case analysis was chosen as study design. The case studies are the historical hospitals in the city centre of Venice, Florence, and Rome. The evaluation of the proposal was carried out through semi-structured interviews with the general managers of the Health Authorities, the medical directors of the selected hospitals and the delegates for the protection and promotion of cultural heritage. The results were analysed using a qualitative model (coding). All respondents welcomed the proposal to launch projects for the humanization of care that foresee the use of the artistic heritage of the historical hospitals to involve patients in the field of health care. Interviewees expressed the desire to invest human and structural resources in the development of these projects. Moreover, directors suggest choosing a specific target with which to start the experimentation and to dispense a specific training to future engaged social and health personnel. The implementation of projects for the humanization of care using the artistic heritage of historical hospitals can be replicated worldwide where healthcare institutions have a cultural wealth to be handed down, shared and valued.

**Key messages:**

• In the post COVID-19 era, it is strategic to exploit artistic heritage owned by the Health Authority for the positive impact in the patient’s experience.

• Artistic heritage claims its role as a health service for supporting patients, caregivers and also health workforce.

